# The Self-Reported Clinical Practice Behaviors of Australian Optometrists as Related to Smoking, Diet and Nutritional Supplementation

**DOI:** 10.1371/journal.pone.0124533

**Published:** 2015-04-17

**Authors:** Laura Elizabeth Downie, Peter Richard Keller

**Affiliations:** 1 Department of Optometry and Vision Sciences, University of Melbourne, Parkville, Victoria, Australia 3010; 2 Macular Research Unit, Centre for Eye Research Australia, East Melbourne, Victoria, Australia 3002; Save Sight Institute, AUSTRALIA

## Abstract

**Objective:**

The primary aim of this study was to examine the self-reported, routine clinical practice behaviors of Australian optometrists with respect to advice regarding smoking, diet and nutritional supplementation. The study also sought to assess the potential influence of practitioner age, gender, practice location (major city versus regional), therapeutic-endorsement status and personal nutritional supplementation habits upon management practices in these areas.

**Methods:**

A survey was electronically distributed to Australian optometrists (n = 4,242). Respondents anonymously provided information about their personal demographics and lifestyle behaviors (i.e., age, gender, practice location, therapeutic-endorsement status, smoking status, nutritional supplement intake) and routine patient management practices with respect to advice across three domains: smoking, diet and nutritional supplementation. Multivariate logistic regression analyses were performed to assess for potential effects of the listed factors on practitioner behavior.

**Results:**

A total of 283 completed surveys were received (completed survey response rate: 6.7%). Fewer than half of respondents indicated routinely asking their patients about smoking status. Younger practitioners were significantly (p < 0.05) less likely to enquire about patients’ smoking behaviors, but this did not extend to counseling for smoking cessation. Almost two-thirds of respondents indicated routinely counseling patients about diet. About half of practitioners specified routinely asking their patients about nutritional supplement intake; this form of questioning was significantly more likely if the respondent was female (p < 0.05). Practitioners who recommended nutritional supplements most commonly did so for age-related macular degeneration (91.2%) and dry eye disease (63.9%). The primary source of evidence used to guide practitioners’ nutrition-related patient management was reported to be peer-reviewed publications.

**Conclusions:**

These findings demonstrate that there are no clear predictors of practitioner behavior across the three domains. Overall, this study suggests that there is scope for Australian optometrists to improve their routine engagement by questioning patients, as well as providing evidence-based clinical advice, about smoking status, diet and nutritional supplement behaviors, being key modifiable lifestyle risk factors with long-term implications for eye health.

## Introduction

Cigarette smoking, diet and nutritional supplementation are major modifiable lifestyle factors that can strongly influence the long-term risk of sight-threatening ocular pathology. Smoking has been causally linked to degenerative eye conditions including cataract[1,2] and age-related macular degeneration (AMD)[3,4], which are both leading causes of vision impairment worldwide. While systemic morbidities associated with smoking, such as lung cancer, cardiovascular disease and stroke, are relatively well known to the general public, associations with eye disease are less widely recognized.[5] In recognition of this, over the past ten years, television- and internet-based advertising campaigns and graphic warnings on cigarette packaging in Australia have sought to improve public awareness about the incident risk of blindness with cigarette smoking. Such interventions have been associated with increased call rates to smoking quit-lines[6] and therefore enhanced awareness is considered of value for altering smoking behaviors.

In addition to smoking, nutrition represents another important potential avenue for modifying a patient’s longer-term risk of eye disease.[7,8] Observational studies have shown an association between diets that are rich in specific nutrients, in particular the xanthophyll carotenoids (zeaxanthin and lutein) and omega-3 essential fatty acids (EFAs), and a reduced risk of sight-threatening late-stage AMD.[9–12] Furthermore, patients who are identified through ophthalmic examination as being at higher risk of developing late AMD may also benefit from specific formulations of antioxidant vitamins and minerals.[13,14] The consumption of high-dose antioxidant supplements is however, not without its own potential risks. For instance, beta-carotene supplementation (dosed at 20 to 30 mg/day) in current smokers or asbestos workers has been linked to an increased risk of lung and stomach cancer.[15] Health care providers therefore play an important role in advising patients about the benefits versus risks of nutritional interventions that may affect their eye, or general, health status.

As the major providers of primary eye care, optometrists are ideally positioned, as part of the health care team, to provide patient counseling with regard to these modifiable risk factors for eye disease.[16–18] In recognition of the important public health role that optometrists provide in these areas, there has been interest in understanding current optometric practices in these domains. Previous studies, which have primarily focused upon self-reported patient management in relation to smoking counseling, have examined the practices of eye care providers in the United Kingdom (UK),[19,20] United States[21] and Canada.[22] Overall, these survey-based investigations reported considerable scope for optometrists in these demographics to be more actively involved in targeted smoking cessation. In relation to diet and nutritional supplementation, only one study has considered optometric clinical management in these areas; this study reported a need to improve awareness among UK optometrists regarding the research evidence underpinning the use of nutritional supplements for AMD.[19] To date, there has not been any research undertaken to examine these practice behaviors in optometrists outside the northern hemisphere. Furthermore, none of these earlier studies considered whether practitioners’ clinical management decisions were influenced by demographic factors or their personal nutritional habits.

The primary aim of this study was to examine the self-reported, routine clinical practice behaviors of Australian optometrists with respect to smoking, diet and nutritional supplementation. The potential influence(s) of practitioners’ age, gender, practice location, therapeutic-endorsement status and personal habits for nutritional supplementation were also examined.

## Materials and Methods

### Participants

A link to a web-based survey was distributed via email to the membership list of Optometry Australia (OA, n = 4,242) in November 2013. OA membership is currently held by approximately 93 percent of registered Australian optometrists (personal communication, National Professional Services Manager, Optometry Australia, October 2013). The survey was designed to assess both the self-reported personal smoking and nutrition-related attitudes and behaviors of optometrists (these findings have been recently reported [23]) and the recommendations made in relation to smoking cessation, diet and nutritional supplementation to patients receiving optometric care (these findings are presented in this manuscript). The project was reviewed and approved by the University of Melbourne Human Research Ethics Committee (Health Sciences sub-committee ID #1340765). A written statement on the first page of the web-link informed potential participants that their electronic submission of the survey implied their consent to participate and that the survey should take up to 10 minutes to complete. Participants were assured that all responses were anonymous and that confidentiality would be strictly maintained.

### Survey design

SurveyMonkey online software was used to host the survey, which comprised of 45 questions; the survey is provided as supplementary material (S1 File). The survey questions were modeled on a previous survey of practitioner behaviors relating to the diagnosis and management of patients with dry eye disease.[24] Respondents were forced to progress through the questions within the survey without reviewing or altering responses on previous pages. The relevant areas of nutrition- and smoking-related patient management behaviors that were investigated were:

- practitioner demographics and lifestyle behaviors: age, gender, practice location (by Australian state and geographic location), therapeutic endorsement status, smoking status, frequency of nutritional supplement intake over the past year;
- patient management: (i) smoking: routine smoking questioning (forced-choice: yes/no response), advice about smoking behaviors (forced-choice: yes/no response) and the rationale for not making recommendations with regard to smoking cessation (open-ended text box), (ii) diet and nutritional supplementation: routine questioning of patients with regard to diet and nutritional supplement intake (forced-choice: yes/no response) and practitioner-driven recommendations regarding nutritional supplements (open-ended text box), and (iii) information and evidence base: sources used by respondents to guide patient care in relation to nutritional supplementation (forced-choice, ranked selection of the three most important sources from the following options: sales representative/marking information, recommendations from colleagues, personal experience with vitamins and supplements, personal clinical impression of effect(s) on patients, non-peer reviewed journal articles, peer-reviewed journal article, meta-analyses of clinical trials and other, with an open-ended text box).

The survey was pilot-tested by two health practitioners (a pharmacist and an optometrist), who were not involved in the creation of the survey, to assess the clarity of the questions and the length of time required to complete the survey.

### Data analyses

Statistical analyses were performed using GraphPad Prism (Version 5.0 for Mac, GraphPad Software Inc., San Jose, CA, USA) and the Statistical Package for the Social Sciences (IBM SPSS, Version 21.0, Armonk, NY, USA). Graphical plots were created using GraphPad Prism. Descriptive statistics were used to analyze practitioner demographics and lifestyle behaviors, patient management (for smoking, diet and nutritional supplementation) and the sources of information and evidence used by practitioners to guide clinical decision-making. Univariate and multivariate logistic regression analyses were performed to assess for potential effects of practitioner age, gender, practice location (major city versus regional), therapeutic endorsement status and personal nutritional supplement intake on patient management. Chi-squared tests were used to compare data consisting of proportions of respondents; an alpha of 0.05 was adopted for statistical significance. Free text responses were used to generate ‘word clouds’, consisting of an image composed of words in which the relative size of the word indicates the frequency in the source text (http://www.wordle.net).

## Results

### Practitioner demographics and lifestyle behaviors

Survey responses were received from 379 optometrists (response rate: 8.9%) over two weeks beginning 4 November 2013. Data from surveys completed in their entirety (n = 283; response rate for completed surveys: 6.7%) were used for analyses. The mean (± SEM) taken by these respondents to complete the survey was 11.8 (± 0.50) minutes.

Respondents varied in age and optometric practice experience (Table 1); 42.4% of practitioners indicated being between 20 and 29 years of age, with the most common age bracket being from 40 to 49 years (26.9% of respondents). A diverse range of optometric experience was evident (range of experience: 1–43 years); the mean (± SEM) years of optometric experience was 18.9 (± 0.90) years.

**Table 1:** Summary of the age and years of optometric experience of the survey respondents.

**Age (years)**	**20–29**	**30–39**	**40–49**	**50–59**	**60+**
% respondents	17.7	24.7	26.9	23.3	7.4
**Time in optometric practice (years)**	**1–10**	**11–20**	**21–30**	**31–40**	**41+**
% respondents	32.1	23.7	25.8	18.0	0.4

The gender split of respondents was: females (56.2%, n = 159) and males (43.8%, n = 124). Responses were received from optometrists practicing in all Australian states and territories. Based upon postcode, most respondents practiced in a major city (71.0%), with the remainder (29.0%) in regional locations (sub-categorized as inner-regional (21.0%), outer-regional (7.6%) or remote (0.4%) locations as defined by the Australian Standard Geographical Classification—Remoteness Area system, 2010 [25]). Sixty-four percent of respondents reported primarily consulting in an independent optometry practice, with 22.3% in a corporate setting and the remainder (13.7%) in alternate practice modalities (e.g., academia, ophthalmology practice, public health clinic). The majority (64.0%) indicated holding current endorsement to prescribe scheduled ocular therapeutic medications.

In relation to practitioners’ own lifestyle behaviors, only one-percent (n = 3) indicated being ‘current’ smokers, herein defined as a person who smokes more than one cigarette per day, 1 cigar per week or chews 30 grams of chewing tobacco for a month, for at least the past year. Almost one in seven (13.3%, n = 38) practitioners indicated having a prior smoking history. Three-quarters of practitioners responded positively to having taken nutritional supplementation over the past year; there was no significant difference in nutritional supplement consumption between genders (p > 0.05). More detailed analyses of the practitioner demographics and personal nutrition-related attitudes and behaviors derived from this survey, which are distinct from the scope of the present paper, are available in a recent publication.[23]

### Patient management

#### Smoking

Overall less than half of respondents (47.4%) indicated routinely asking their patients whether they smoke(d). No significant association was observed for any of gender (male versus female practitioners), practice location (major city versus regional) or therapeutic- versus non-therapeutically-endorsed optometrists (p > 0.05) and these variables were not included in the multivariate analysis (Table 2).

**Table 2:** Predictors of practitioner behavior in relation to asking about patient smoking status.

	Univariate model	Multivariate model	
Variable	p value	p value	Adjusted OR (95% CI)
Own smoking status	0.383		
Taken dietary supplements in past year	0.017*	0.090	
Own annual expenditure on dietary supplements	0.282		
Gender	0.171		
Age	0.034*	0.016*	1.30 (1.05 to 1.61)
Therapeutic endorsement status	0.230		
Years in practice	0.116		
Practice location	0.632		

* indicates a statistically significant difference (p < 0.05).

Only those variables found to be statistically significant by multiple univariate modeling were included in the subsequent multivariate modeling. Smoking status was collapsed to become dichotomous, i.e., ever or never.

A significant inverse association was found between practitioner age and the likelihood of routinely asking patients about their smoking behaviors (Tables 2 and 3). Younger practitioners were significantly less likely to ask their patients whether they smoke than older practitioners (p = 0.016; adjusted odds ratio, OR 1.30, 95% CI: 1.05 to 1.61).

**Table 3:** Summary of the proportion of survey respondents in each age bracket who indicated routinely asking their patients about smoking behaviors.

Age (years)	20–29	30–39	40–49	50–59	60+
% respondents	34.0*	38.0	48.7	56.1	57.4

* indicates a significant difference (p < 0.05) compared with respondents in all other age brackets.

Of the respondents who indicated questioning their patients about smoking status, most (80.2%) responded positively to providing recommendations about smoking behavior and this association was highly significant (p < 0.001). Multivariate analyses showed no significant difference in approach between male versus female practitioners (p > 0.05), those practicing in urban versus regional areas (p > 0.05) or therapeutic- versus non-therapeutically-endorsed optometrists (p > 0.05). Unlike the findings relating to smoking questioning, no significant association was found between practitioner age and the likelihood of providing counseling with regard to smoking cessation (Table 4).

**Table 4:** Predictors of practitioner behavior in relation to providing advice to patients about smoking cessation.

	Univariate model
Variable	p value
Own smoking status	0.982
Taken dietary supplements in past year	0.979
Own annual expenditure on dietary supplements	0.378
Gender	0.178
Age	0.178
Therapeutic endorsement status	0.565
Years in practice	0.366
Practice location	0.465
Asks about patient smoking status	< 0.001*

Except for whether a practitioner asks about smoking status, no variable was found to be statistically significant (*) by multiple univariate modeling and no multivariate modeling was applied.

**Fig 1:**
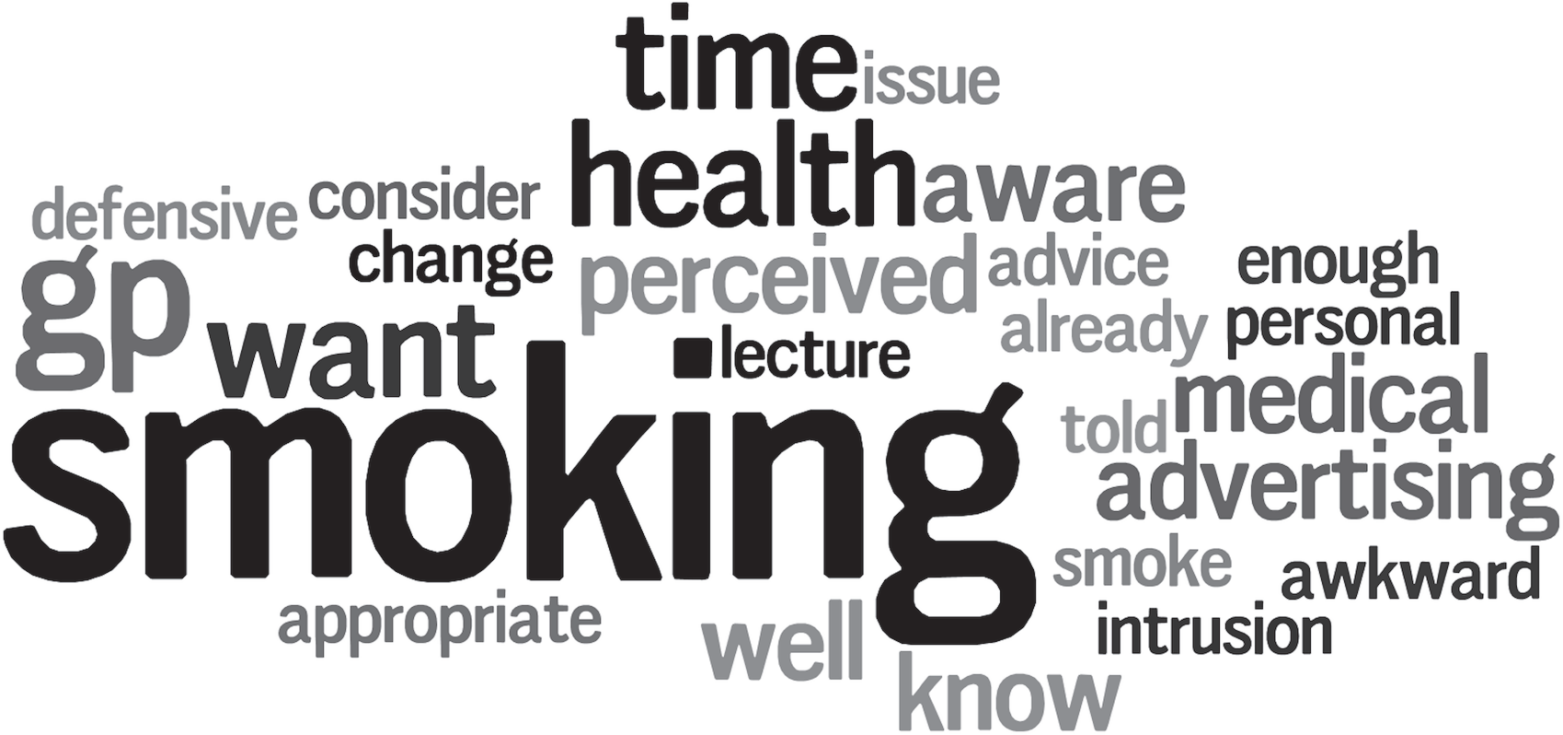
A ‘word cloud’ of the 25 most frequently reported words generated from free-text responses from optometrists (n = 52) to a question asking for the reason(s) why they do not counsel patients regarding smoking behaviors.

Respondents who indicated that they did not routinely make recommendations to patients with regard to smoking behavior were invited to comment on the reason(s) for this in a free-text commentary box. Fig 1 shows a ‘word cloud’ visual representation of these free-text responses. As represented by the size of the lettering, many respondents considered smoking counseling to be a medical issue that was the responsibility of the patient’s general medical practitioner (GP). Other common responses included a lack of practitioner time, sufficient patient awareness from advertising campaigns about the health risks associated with smoking and/or that this type of questioning was too personal or intrusive.

#### Diet and nutritional supplementation

Less than two-thirds of optometrists (62.2%, n = 176) indicated routinely counseling their patients with regard to diet. No significant association was found between providing advice regarding diet and practitioner gender (p > 0.05), practice location (p > 0.05) or ocular therapeutic endorsement status (p > 0.05). Although univariate regression analysis suggested older practitioners and those personally taking nutritional supplements over the past 12 months were more likely to provide dietary recommendations to patients, this was not confirmed by multivariate analysis (Table 5).

**Table 5:** Predictors of practitioner behavior in relation to advising patients about their diet.

	Univariate model	Multivariate model
Variable	p value	p value
Own smoking status	0.659	
Taken dietary supplements in past year	0.050*	0.086
Own annual expenditure on dietary supplements	0.123	
Gender	0.827	
Age	0.050*	0.662
Therapeutic endorsement status	0.266	
Years in practice	0.004*	0.485
Practice location	0.590	

* indicates a statistically significant difference (p < 0.05).

Only those variables found to be statistically significant by multiple univariate modeling were included in the subsequent multivariate modeling. No variable was found to be statistically significant after multivariate modeling was applied.

Slightly more than half of respondents (54.8%, n = 155) specified routinely asking their patients if they were taking nutritional supplements. As shown in Table 6, no significant differences were observed in relation to practitioner age (p > 0.05) or ocular therapeutic endorsement status (p > 0.05). Practitioner gender was the only independent variable found to be associated with asking about taking nutritional supplements (p = 0.017) with female practitioners significantly more likely than male practitioners to enquire about nutritional supplement intake (unadjusted OR 1.78, 95% CI: 1.11 to 2.86).

**Table 6:** Predictors of practitioner behavior in relation to asking patients about their use of nutritional supplements.

	Univariate model	
Variable	p value	Unadjusted OR (95% CI)
Own smoking status	0.406	
Taken dietary supplements in past year	0.606	
Own annual expenditure on dietary supplements	0.632	
Gender (female)	0.017*	1.78 (1.11 to 2.86)
Age	0.514	
Therapeutic endorsement status	0.952	
Years in practice	0.552	
Practice location	0.774	

* indicates a statistically significant difference (p < 0.05).

Only one variable was found to be statistically significant by multiple univariate modeling and no multivariate modeling was applied.

Respondents were also asked whether they routinely suggest to their patients to use nutritional supplements and to specify the medical conditions underlying these recommendations. Positive recommendations for nutritional supplements were made by 80.2% (n = 227) of practitioners. Unlike the association between gender and asking about supplement use, there was no significant association between a practitioner’s gender and whether they provided advice about nutritional supplementation use to their patients (Table 7). A negative association was found between practitioner age and providing advice about supplement use, with younger practitioners more likely to provide such advice (p = 0.005; unadjusted OR 1.43, 95% CI: 1.11 to 1.84).

**Table 7:** Predictors of practitioner behavior in relation to advising patients about the use of nutritional supplements.

	Univariate model	
Variable	p value	Unadjusted OR (95% CI)
Own smoking status	0.638	
Taken dietary supplements in past year	0.615	
Own annual expenditure on dietary supplements	0.195	
Gender	0.459	
Age	0.005*	1.43 (1.11 to 1.84)
Therapeutic endorsement status	0.981	
Years in practice	0. 11	
Practice location	0.290	

* indicates a statistically significant difference (p < 0.05).

Only one variable was found to be statistically significant by multiple univariate modeling and therefore no multivariate modeling was applied.

The three most common conditions that these practitioners reported to promote nutritional supplementation for were age-related macular degeneration (AMD, 91.2%), dry eye disease (63.9%) and eyelid twitching (3.5%); other responses are detailed in Table 8. Of the supplements recommended for AMD, the most common were various forms of high-dose antioxidants (89.8%), and omega-3 essential fatty acids (EFAs, 8.5%). Most practitioners advocating nutritional supplementation for dry eye disease recommended omega-3 EFAs (88.7%), with a few suggesting Vitamin E (2.7%) supplements.

**Table 8:** Summary of the proportion of survey respondents, of those who routinely recommended nutritional supplements to patients, who nominated each ocular or general health condition.

Medical condition	% Respondents
AMD	91.2
Dry eye disease	63.9
Other	7.1
Eyelid twitching	3.5
Diabetic retinopathy	1.3
Anemia	1.3
Viral ocular infection	1.3

AMD, age-related macular degeneration; ‘Other’ includes: arthritis, cataract, glare, glaucoma, hordeolum, hypercholesterolemia, myopia, nyctalopia, osteoporosis, scurvy and skin abnormalities.

#### Information and evidence base

**Fig 2:**
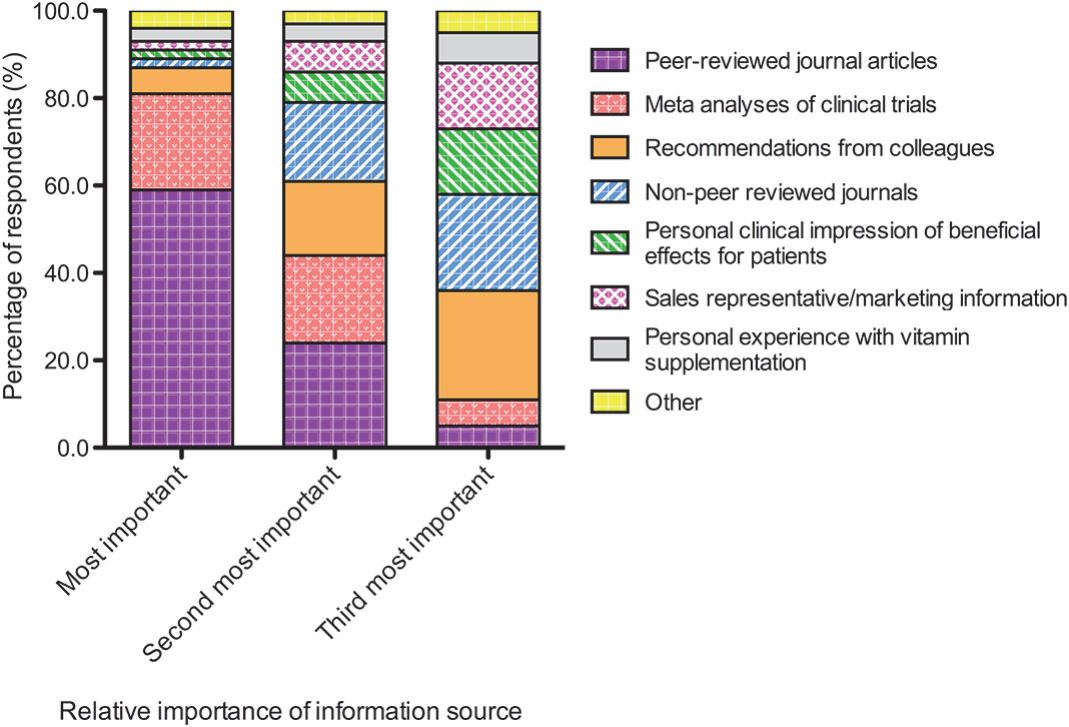
Stacked bar chart showing the proportion of survey respondents who ranked, in order of importance, the various sources of information or evidence that they used to guide their clinical decision making for recommending nutritional supplementation.

Respondents indicated using a range of different resources to guide their clinical decision-making in relation to recommending nutritional supplements to their patients (Fig 2). Overall, the three most important influences selected by respondents were peer-reviewed journals (88.5%), recommendations from colleagues (48.3%) and meta-analyses of clinical trials (48.1%). Other sources that were frequently selected were non-peer reviewed journal articles (43.4%), sales representative/marketing information (24.0%) and the personal clinical impression of beneficial effects for patients (24.0%).

## Discussion

This study has analyzed the self-reported clinical management practices of Australian optometrists with respect to smoking, diet and nutritional supplementation. This is the first study to have assessed such practices in optometrists within the southern hemisphere. No previous studies have specifically examined the potential influence of practitioner-derived factors (including age, gender, therapeutic endorsement status, practice location and personal nutritional supplement intake) on patient care in these areas. Furthermore, we examined optometrists’ self-reported behaviors regarding the sources of information and evidence that they use to guide clinical decision marking.

A major finding of this study is that fewer than half of respondents indicated routinely enquiring about their patients’ smoking status. Tobacco smoking has significant adverse effects upon eye health;[26] in particular, smoking is the most important modifiable risk factor for age-related macular degeneration (AMD), being the leading cause of permanent vision impairment in persons aged 50 years or over in developed countries.[27] Smoking at least doubles the risk of developing AMD;[28] a direct association also exists between the number of cigarettes smoked over time and the risk of late-stage AMD, being the vision-threatening form of the disease.[29] Importantly, a degree of reversibility exists to these detrimental effects of smoking on retinal health. The risk of developing AMD decreases substantially following smoking cessation and is also lowered by a reduction in smoking intensity.[30,31] The risk of a former smoker, who has not smoked for 20 years, developing AMD is reported to be comparable to a person who has never smoked.[29] Such risk reduction has been proposed to potentially provide a highly valuable health message to encourage smoking cessation.[30] A specific demonstration of the benefit of quitting smoking for an individual patient can be achieved through using an accurate online AMD ‘risk calculator’ which can illustrates the reduction in risk estimate for late-stage AMD, based upon smoking status.[32]

We acknowledge the limitation of smoking questioning with respect to smoking deception, involving patients failing to self-report as smokers, and the potential misclassification of smoking status.[33] However, that the majority of survey respondents indicated a lack of routine enquiry about their patients’ smoking behaviors raises a significant public health concern. Moreover, of the practitioners who undertook basic smoking questioning, one in five indicated that they would not routinely act upon this information to initiate counseling to quit smoking. Similar findings were reported in a pilot study conducted in the United States; most eye care providers indicated that they rarely or only periodically questioned their patients about smoking status, willingness to stop smoking or made recommendations to cease smoking.[21] Comparable deficiencies in smoking-related care practices by optometrists have been reported in recent studies conducted in the UK [19,20] and Canada.[22] Enhanced primary care recommendations to promote smoking cessation would be predicted to translate into immense health and economic benefits. It has been estimated that if cigarette smoking was halved in Australia, cases of AMD would reduce by one-sixth, with savings to the economy in the order of $250 million per year.[34]

Notably, younger optometrists were significantly less likely to ask about a patient’s smoking status but were more likely to provide advice on the use of dietary supplements. A recent European study found age-dependent differences in the knowledge of medical physicians relating to pharmacotherapy for tobacco cessation; physicians aged 50 years or over demonstrated higher knowledge scores than younger doctors.[35]. It is possible that, as non-smokers themselves and being younger than their patients, younger optometrists were more reluctant to question patients on this topic, although they were no less likely to provide advice on smoking cessation.

In the present study the two most frequent justifications given by respondents for not providing patient counseling to quit smoking were a perception that this form of medical care was primarily the responsibility of the general medical practitioner (GP) and/or a lack of sufficient consultation time to administer such advice. Inadequate training to prepare eye care providers to deliver smoking cessation assistance to patients has been reported as a major barrier to initiating smoking-related counseling.[21] For GPs, one hour training sessions have been shown to be valuable for improving the incidence of patient questioning about smoking behaviors and for enhancing the provision of quit-smoking advice;[36] similar programs may be of value for Australian optometrists. A perceived lack of time to provide smoking cessation advice could potentially be overcome through the development of a clinical tool for optometrists to capture essential information regarding smoking status. The information provided by patients could then be used as a basis for a discussion about smoking behavior and developing an appropriate care plan, in association with the patient’s GP and a smoking cessation specialist. Indeed, recommendations from health care providers have been shown to be strongly influential in supporting smoking cessation, with positive effects on quit rates.[37,38] Even a short period of patient counseling, of less than three minutes in duration, has been shown to increase smoking cessation rates by up to 30%.[37]

In addition to smoking, nutrition is another potentially modifiable lifestyle factor that can alter a patient’s long-term risk of developing sight-threatening eye disease.[7,8] A wealth of epidemiological data confirm the possible benefit of diets rich in zeaxanthin, lutein and omega-3 EFAs (docosahexaenoic acid, DHA and eicosapentaenoic acid, EPA) for reducing the risk of late-stage AMD.[9–12] Lutein and zeaxanthin, which are found in high concentration in the human macula, cannot be generated *in vivo* and therefore must derive from dietary sources. Foods rich in these carotenoids include kale, spinach, peas, pumpkin and eggs. The xanthophyll carotenoids have been implicated in a number of essential physiological functions in the retina, including anti-oxidant protection and the filtration of short-wavelength light.[39] Omega-3 EFAs, which must also be obtained dietary sources, are found in relative abundance in oily fish, such as salmon, mackerel and sardines. In the context of influencing retinal integrity, DHA is a major structural component of retinal membranes, with tissue DHA levels affecting retinal cell signaling mechanisms involved in phototransduction.[40] DHA and EPA may also impart retinoprotective effects through modulating cellular differentiation and survival.[41] Such biological functions provide the scientific rationale for lutein, zeaxanthin and omega-3 EFAs having a potential retinal protective effect in AMD. These roles, combined with the observational evidence relating to the apparent benefit of particular dietary patterns, [9–12] were the basis for clinical trials that have sought to assess whether high-dose nutritional supplements are of value for slowing AMD progression. In particular, two large, multi-center, randomized, controlled clinical trials (the Age-Related Eye Disease Study (AREDS)[13] and AREDS2,[14]) evaluated the safety and efficacy of high-dose antioxidant dietary supplements for slowing the progression to advanced AMD. Through post-hoc sub-group analyses, AREDS showed that a combination of high-dose antioxidant vitamins and nutrients, could reduce the risk of progression from intermediate to late AMD by 25% (i.e., from 28% to 20%) over a five year period.[13]

Such interactions between nutrition and eye health are clearly relevant to the delivery of primary eye care by optometrists. Specifically, optometrists are well positioned to initiate conversations about their patients’ dietary habits and to advise with regard to whether certain foods and/or nutritional supplements could be beneficial, or otherwise, to ocular health. As a starting point, this could be in the form of simple advice that is supported by the epidemiological evidence, such as that promoted the Macular Disease Foundation Australia, to encourage a healthy well-balanced diet that incorporates the consumption of dark green leafy vegetables daily and oily fish two to three times per week. More advanced, nutrition-focused healthcare could then be achieved through appropriate co-management with GPs and other health professionals, such as dieticians, to achieve desirable lifestyle changes.

In the present survey, it was found that almost 40 percent of respondents would not routinely counsel patients about their diet. While it could be suggested that this is due, at least in part, to a relatively weak understanding of the importance of nutrition for eye health, another potential contributory factor is that dietary assessment is perceived as complex and beyond the scope of routine practice for optometrists. There are currently no simple quantitative clinical tools available to eye care providers to assess the key aspects of diet that may be important for eye health. Addressing this issue, along with providing associated education programs, may enable such discussions about diet and nutrition to be implemented more readily into optometric practice.

The two eye conditions that practitioners most frequently recommended nutritional supplements for were AMD (91.2%) and dry eye disease (63.9%). The current survey did not seek to assess participants’ knowledge of the possible benefit of nutritional supplementation relative to disease severity, as was undertaken in relation to AMD in a UK demographic.[19] However, our findings relating to recommendations for omega-3 EFA supplements to treat dry eye disease are noteworthy in the context of assessing practitioner understanding of the available research evidence. While no systematic reviews are currently available on this topic, several recent proof-of-concept, randomized, controlled clinical trials have reported short-term clinical improvements with oral omega-3 EFA supplements in patients with dry eye disease.[42–45] Omega-3 EFA supplements show promise as a dry eye therapy, however further research is needed to guide clinical recommendations with regard to the optimal dose, composition and specific indications for use.[24,46]

Regarding the information and evidence used by respondents to guide their clinical decision-making for recommending nutritional supplements to patients, the most common response was peer-reviewed journal articles (88.5%). These findings contrast to a recent survey, conducted by our research group on the optometric management of dry eye disease, which reported that less than one quarter of respondents identified using peer-reviewed resources to guide their clinical management.[24] The current data also differ from findings in a study of UK optometrists, in which the majority of respondents indicated using professional magazines to guide practice recommendations for diet and nutritional supplementation.[19] In the present survey, non-peer reviewed journals were selected as one of the three most important information sources by less than half (43.4%) of respondents. The self-reported use of peer-reviewed journal articles by such a high percentage of respondents in this survey is encouraging. Furthermore, almost half of the survey respondents indicated using meta-analyses in their top three sources of information and evidence. Such behaviors imply knowledge of the importance of accessing these types of resources to guide clinical decisions to deliver evidence-based care. We did not however, attempt to assess clinicians’ depth of understanding with regard to the interpretation of research studies; indeed, complex trials such as AREDS and AREDS2 are recognized to require a thorough knowledge of clinical trial design for accurate interpretation and application to practice.[7]

We acknowledge some limitations to the present study. With a survey response rate of 8.9% there is the potential for the findings to be influenced by selection bias. In particular, a tendency for individuals who are more engaged with the topic being more likely to provide their response. Our finding that just under half of respondents would routinely enquire about smoking status and nutrition may be an overestimation when applied to the wider Australian optometric population. Indeed, significantly lower rates of smoking questioning have been reported in studies from other demographics.[19,22] Although we are unable to determine whether this difference is due to a selection bias or a greater appreciation of the importance of smoking cessation by Australia optometrists, these findings, taken together suggest that there is still scope for improvement in this area.

The proportion of respondents who reported holding therapeutic endorsement (64%) was somewhat higher than in the general population of Australian optometrists, at 37%.[47] Nonetheless, the sub-group analyses for smoking and diet patient management practices did not show any significant differences in self-reported behaviors between therapeutically-endorsed and non-therapeutically endorsed practitioners. Inherent with a conservative response rate is the potential for the findings to not necessarily be fully representative of the broader Australian optometric profession. We also acknowledge that the data are self-reported, relying upon factors such as the truthfulness of the responses, the respondents’ introspective abilities and their comprehension and interpretation of the survey questions.

Nevertheless, the population sample accurately reflects both the age and gender distribution of the Australian optometric workforce[48] and is not dissimilar to other surveys in the literature that have investigated optometric practice behaviors.[22,24] Furthermore, the relative distribution of urban versus regional practitioners aligns with the documented Australian workforce coverage, which is concentrated in metropolitan areas; in 2012, approximately three quarters of optometrists were practicing in major cities.[49] Distribution of the survey via an electronic medium enabled responses from a diverse range of practitioners, differing in age, level of optometric practice experience and geographic location. Furthermore, the study design enabled for the assessment of the effects of inherent practitioner factors (including age, gender, therapeutic endorsement status and personal nutritional supplement intake) on patient management approaches for smoking, diet and nutritional supplementation.

## Conclusions

The present study provides a valuable insight into the current self-reported practice behaviors of Australian optometrists, as related to smoking and nutrition. The findings suggest that many practitioners do not routinely ask their patients about smoking status, nor do they provide counseling with regard to smoking cessation; younger optometrists (20 to 29 years of age) were less likely to enquire about smoking behaviors than older practitioners. Overall, optometrists tended to provide patient advice in relation to diet and nutritional supplementation slightly more regularly than for smoking. Almost two-thirds of respondents indicated routinely counseling patients about their diet; practitioners who consumed nutritional supplements themselves were no more likely to discuss the use of dietary supplements with their patients. These findings highlight scope to improve patient-related guidance in relation to smoking, diet and nutritional supplementation in Australian optometric practice.

## Supporting Information

S1 File Nutritional supplements survey to Australian optometrists. Hard copy of the survey that was electronically distributed to participants.
(PDF)
