# Supplementary material for: The Self-Reported Clinical Practice Behaviors of Australian Optometrists as Related to Smoking, Diet and Nutritional Supplementation
Source: PLoS One. 2015 Apr 17;10(4):e0124533. doi: 10.1371/journal.pone.0124533 (PMC4401759; doi:10.1371/journal.pone.0124533)
Supplement: S1 File — Hard copy of the survey that was electronically distributed to participants. (PDF) [file pone.0124533.s001.pdf]

Nutritional Supplements Survey to Australian Optometrists

Practitioner Behaviours and Lifestyle

Dear Colleague,

The purpose of this survey is to assess the use of nutritional supplements by Australian optometrists and the recommendations made to patients in relation to nutrition.

Your return of this survey implies your consent to participate in this research. The survey is anonymous and your confidentiality will be strictly maintained.

Your contribution to this research is appreciated. The survey should take about 7-10 minutes to complete.

The study has been approved by the University of Melbourne Human Research Ethics Committee (Ethics ID: 1340765).

For further information, please contact Dr Laura Downie (email: ldownie@unimelb.edu.au, phone: 03 9035 3043).

Should you have any concerns about the conduct of this research project, you can contact the Executive Officer, Human Research Ethics, The University of Melbourne, phone: 8344 2073.

Thank you for your support.

Dr Laura Downie and A/Prof Peter Keller
Department of Optometry and Vision Sciences
The University of Melbourne

***Do you eat what you would consider to be a healthy, balanced diet?**

- ○ Yes
- ○ No

***Are you currently pregnant or breastfeeding?**

- ○ Yes
- ○ No

***Are you currently planning a pregnancy?**

- ○ Yes
- ○ No

***What would be your estimated expenditure on beauty products (including all grooming products) over the past 12 months (including products used by you, but purchased by others)?**

$ [ ]

Smoking Behaviours

Definition: A current smoker is one (or more) of: smoking more than one cigarette per day, 1 cigar per week or 30 grams of chewing tobacco for a month, for at least the past year.

*** Do you currently smoke?**

- Yes
- No

Definition of:

- No: “Never smoked” is less than one cigarette per day, 1 cigar per week or 30 grams of chewing tobacco per month, for no more than one year;

- Yes: “Previously smoked” is no smoking for at least one year, but previously either one of: one or more cigarettes per day, 1 cigar per week or 30 grams of chewing tobacco per month.

*** Have you ever smoked?**

- Yes
- No

Nutritional Supplements - Part 1

For the purpose of this survey, a 'nutritional supplement' describes any non-prescription vitamin or dietary supplement.

*** Over the past twelve months, how frequently have you taken nutritional supplements?**

- Regularly
- Sometimes
- Rarely
- Never

Nutritional Supplements - Never Used

***In order of importance, select which three factor(s) underlie your decision to NOT routinely take nutritional supplements?**

Most Important Factor	☐ ▼
Second Most Important Factor	☐ ▼
Third Most Important Factor	☐ ▼

Other (please specify)

Nutritional Supplements - Part 1b

***What would be your estimated expenditure on nutritional supplements over the past 12 months (including supplements consumed by you, but purchased by others)?**

$

Indicate whether your decision to take nutritional supplements was a result of the recommendations of your: (Check all that apply)

- ☐ General medical practitioner
- ☐ Other (specialist) medical practitioner
- ☐ Optometric colleague
- ☐ Pharmacist / chemist
- ☐ Friends / family
- ☐ Health shop assistant
- ☐ Naturopath / herbalist / homeopath
- ☐ Self

Other (please specify)

Practitioner Behaviours - Nutritional Supplements - Supplement Types

Which of the following nutritional supplements have you consumed over the past 12 months? (Check all that apply)

- ○ Multivitamin

- Calcium
- Fish Oil / Omega-3s
- Folate
- Glucosamine
- Magnesium
- Echinacea
- Zinc
- Iron
- St John's Wort
- Coenzyme Q10
- Carotenoids (e.g., lutein, zeaxanthin)
- Vitamin B12
- Vitamin C
- Vitamin D

- Vitamin E

- Other

Other (please specify)

Practitioner Behaviours - Nutritional Supplements - Conditions and Influenc...

For each positive response in the previous question, detail which condition you are taking the nutritional supplement for:

[Q11]

[Q12]

[Q13]

[Q14]

[Q15]

[Q16]

[Q17]

[Q18]

[Q19]

[Q20]

[Q21]

[Q22]

[Q23]

[Q24]

[Q25]

[Q26]

[Q27]

Nutritional Supplements Survey to Australian Optometrists

For each of the nutritional supplements that you selected, indicate whether your decision to take this medicine was a result of the recommendations of your: (Check all that apply)

	Noone (self-decided)	General medical practitioner	Other medical practitioner	Optometric colleague	Pharmacist / Chemist	Friends / Family	Health shop assistant	Naturopath / Herbalist / Homeopath	Other
[Q11]	☐	☐	☐	☐	☐	☐	☐	☐	☐
[Q12]	☐	☐	☐	☐	☐	☐	☐	☐	☐
[Q13]	☐	☐	☐	☐	☐	☐	☐	☐	☐
[Q14]	☐	☐	☐	☐	☐	☐	☐	☐	☐
[Q15]	☐	☐	☐	☐	☐	☐	☐	☐	☐
[Q16]	☐	☐	☐	☐	☐	☐	☐	☐	☐
[Q17]	☐	☐	☐	☐	☐	☐	☐	☐	☐
[Q18]	☐	☐	☐	☐	☐	☐	☐	☐	☐
[Q19]	☐	☐	☐	☐	☐	☐	☐	☐	☐
[Q20]	☐	☐	☐	☐	☐	☐	☐	☐	☐
[Q21]	☐	☐	☐	☐	☐	☐	☐	☐	☐
[Q22]	☐	☐	☐	☐	☐	☐	☐	☐	☐
[Q23]	☐	☐	☐	☐	☐	☐	☐	☐	☐
[Q24]	☐	☐	☐	☐	☐	☐	☐	☐	☐
[Q25]	☐	☐	☐	☐	☐	☐	☐	☐	☐
[Q26]	☐	☐	☐	☐	☐	☐	☐	☐	☐
[Q27]	☐	☐	☐	☐	☐	☐	☐	☐	☐

Practitioner Behaviours - Nutritional Supplements - Conditions and Influenc...

Nutritional Supplements Survey to Australian Optometrists

In order of importance, select which three factor(s) underlied your INITIAL decision to take these products.

	MOST Important Factor	SECOND MOST Important Factor	THIRD MOST Important Factor
[Q11]			
[Q12]			
[Q13]			
[Q14]			
[Q15]			
[Q16]			
[Q17]			
[Q18]			
[Q19]			
[Q20]			
[Q21]			
[Q22]			
[Q23]			
[Q24]			
[Q25]			
[Q26]			
[Q27]			

Other (please specify)

In order of importance, select which three factor(s) underlie your ONGOING decision to take these products.

	MOST Important Factor	SECOND MOST Important Factor	THIRD MOST Important Factor
[Q11]			
[Q12]			
[Q13]			
[Q14]			
[Q15]			
[Q16]			
[Q17]			
[Q18]			
[Q19]			
[Q20]			
[Q21]			
[Q22]			
[Q23]			
[Q24]			
[Q25]			
[Q26]			
[Q27]			

Other (please specify)

Patient Management

***Do you routinely ask your patients whether they smoke?**

- Yes
- No

***Do you provide recommendations to your patients with regard to their smoking behaviour?**

- Yes
- No

If No, why not?

***Do you routinely provide recommendations to your patients regarding their diet?**

- Yes
- No

***Do you routinely ask whether your patients take any nutritional supplements?**

- Yes
- No

List any nutritional supplements that you recommend to your patients and the conditions that you would recommend them for:

1. Vitamin / Condition:
2. Vitamin / Condition:
3. Vitamin / Condition:
4. Vitamin / Condition:
5. Vitamin / Condition:

***In order of importance, rank the three sources of information or evidence that you use to guide your decision-making for these recommendations to your patients:**

Most Importance Source

Second Most Important Source

Third Most Important Source

Other (please specify)

Practice and Practitioner Demographics

***What is your gender?**

- Male
- Female

***Indicate your age bracket:**

- 20-29
- 30-39
- 40-49
- 50-59
- 60-69
- 70+

***Were you awarded your optometric qualification in Australia?**

- Yes
- No

***Are you endorsed to prescribe ocular therapeutic drugs?**

- Yes
- No

***For how many years have you been practicing as an optometrist?**

***What is the postcode of your primary practice address?**

***Which of the following best describes your major mode of optometric practice (Check one box only)**

- Corporate practice
- Independent practice (single or multiple owners)
- Academic institution
- Other

Please specify

Nutritional Supplements Survey to Australian Optometrists

***What is your professional scope of practice (Check all that apply)**

- [ ] General primary eye care
- [ ] Contact lenses
- [ ] Behavioural optometry / paediatrics
- [ ] Ocular disease management
- [ ] Low vision
- [ ] Other

Please specify

Thank you for your completing this survey.
